# The Genus *Criodion* (Audinet-Serville, 1833) (Coleoptera, Cerambycidae): First Record for Panama

**DOI:** 10.3897/BDJ.4.e7968

**Published:** 2016-03-07

**Authors:** Alfredo Lanuza-Garay, David Ezequiel Herrera, Margarita Marin, Alonso Santos Murgas

**Affiliations:** ‡ Universidad de Panamá, Centro Regional Universitario de Colón, Departamento de Zoología, Colon, Panama; § Smithsonian Tropical Research Institute, Punta Galeta Marine Laboratory 0843-03092, Panama, Panama; | Universidad de Panamá, Centro Regional Universitario de Colón, Escuela de Biología, Colon, Panama; ¶ Universidad de Panamá; Facultad de Ciencias Naturales Exactas y Tecnología, Museo de Invertebrados G. B. Fairchild, Departamento de Zoología, Panama, Panama

**Keywords:** Sphallotrichina, *
Criodion
*, *
cinereum
*, *
tuberculatum
*

## Abstract

**Background:**

The Cerambycidae are one of the largest beetle families. Cerambycid beetles are found on all continents, but the tropics are extremely rich in this species. The genus *Criodion* (Audinet-Serville, 1833) includes 13 species in the Neotropical Region, two of which occur in Central America. Panama has a high biodiversity, yet a small number of sites have been extensively studied. In this contribution, new distributional data are given for *C. cinereum* (Olivier, 1795) and *C. tuberculatum* Gahan, 1892.

**New information:**

Two species of the genus *Criodion* (Audinet-Serville, 1833) are recorded for first time in Panama, *Criodion cinereum* (Olivier, 1795) and *Criodion tuberculatum* Gahan, 1892. Relevant details are presented for each species.

## Introduction

The Cerambycidae, commonly known as Longhorn beetles, comprise one of the largest and most varied families of the Coleoptera, and though they have been studied for a century, descriptions of new species and new distributional records have accelerated in recent decades (Bezark 2014, Monné 2015). The genus *Criodion* (Audinet-Serville, 1833) currently includes 13 described neotropical species that range from Guatemala to Argentina, eleven of which are distributed in South America, where some are endemics and others are widespread (Martins 2004). We report two new records for the Panamanian fauna: *Criodion cinereum* (Olivier, 1795) and *Criodion tuberculatum* Gahan, 1892.

## Materials and methods

Photographs were taken with a Nikon D7000 camera, Nikon-DX- 18-55mm macro lens. Specimens were studied using a Leica MZ6 stereomicroscope, and their identification was carried out with the aid of the reference collection at the MIUP, and the identification keys of Martins 2004.

Acronyms for the collections consulted during this study are as follows:

**CRUC** Colección de insectos, Escuela de Biología, Universidad de Panamá Centro Regional de Colon;

**MIUP** Museo de Invertebrados Graham Bell Fairchild, Universidad de Panamá.

## Taxon treatments

### Criodion cinereum

(Olivier, 1795)

#### Materials

- **Type status:** Other material. **Occurrence:** occurrenceRemarks: collected in a secondary forest; recordedBy: David Herrera; individualCount: 1; sex: female; lifeStage: Adult; **Taxon:** scientificName: *Criodion cinereum* (Olivier, 1795); acceptedNameUsage: Criodion cinereum; parentNameUsage: Cerambycidae; originalNameUsage: *Prionus cinereus* Olivier 1795; nameAccordingTo: Gahan, C.J. 1892. Notes on Longicorn Coleoptera of the group Cerambycinae, with descriptions of new species. Ann. Mag. Nat. Hist. London 6(9): 24.; namePublishedIn: Monne, M.A. 1993. Catalogue of the Cerambycidae (Coleoptera) of the Western Hemisphere. Part III: Subfamilia Cerambycinae: Tribes Cerambycini, Diorini and Piezocerini. Sao Paulo, Sociedade Brasileira de Entomologia. 52pp.; higherClassification: "Animalia"; "Arthropoda"; "Hexapoda"; "Insecta"; "Coleoptera"; "Cerambycidae"; "Cerambycinae"; "Spallotrichina"; "Criodon"; "Criodon cinereum"; kingdom: Animalia; phylum: Arthropoda; class: Insecta; order: Coleoptera; family: Cerambycidae; genus: Criodion; specificEpithet: cinereum; taxonRank: species; scientificNameAuthorship: (Olivier, 1795); vernacularName: Longhorn beetle; nomenclaturalCode: ICZN; taxonomicStatus: accepted; **Location:** higherGeographyID: TGN: 1001208; higherGeography: Central America, Panama, Colon, Colon, Quebrada Ancha, Curva el Cebo; continent: Central America; country: Panama; countryCode: Pa; stateProvince: Colon; county: Colon; municipality: Quebrada Ancha; locality: Curva el Cebo; verbatimElevation: 154 m; minimumElevationInMeters: 0; maximumElevationInMeters: 154; minimumDistanceAboveSurfaceInMeters: 0; maximumDistanceAboveSurfaceInMeters: 0; verbatimCoordinates: 09°17'08.99"N, 79°44'15.08"W; **Identification:** identificationID: Criodion cinereum; identifiedBy: Alfredo Lanuza-Garay; dateIdentified: 2015-12-10; identificationReferences: "Entomologie, ou histoire naturelle des insectes Olivier 1795", "Cerambycidae Sul Americanos. Martins 2004"; identificationRemarks: Distinguished between *Criodion cinereum* and *C. torticolle* based on the basal antenommere not reaching the anterior margin of the pronotum, pronotal gibbosity scarecely apparent; the prosternal process not exceeding the procoxae and middle femoral apical spines not markedly projected; **Event:** samplingProtocol: manual; eventDate: 2015-10-18; year: 2015; month: 10; day: 18; habitat: Secondary forest; **Record Level:** type: Physicalobject; language: es; rightsHolder: Universidad de Panama; bibliographicCitation: Olivier, A.G. 1795. Entomologie, ou histoire naturelle des insectes, avec leurs caractères génériques et spécifiques, leur description, leur synonymie, et leur figure enluminée. Coléoptères.Desray, Paris 4:1‑519; institutionID: Escuela de Biologia, Centro Regional Universitario de Colon, Universidad de Panama; institutionCode: CRUC; collectionCode: Insects; ownerInstitutionCode: CRUC-UP; basisOfRecord: preserved specimen

#### Distribution

This species has been recorded from Costa Rica, Colombia, Bolivia, Brazil, French Guiana, Peru, Surinam, and Venezuela (Monné 2015). The reported occurrences of *C. cinereum* in Puerto Rico and Paraguay are doubtful.

#### Notes

**Figure 1.**
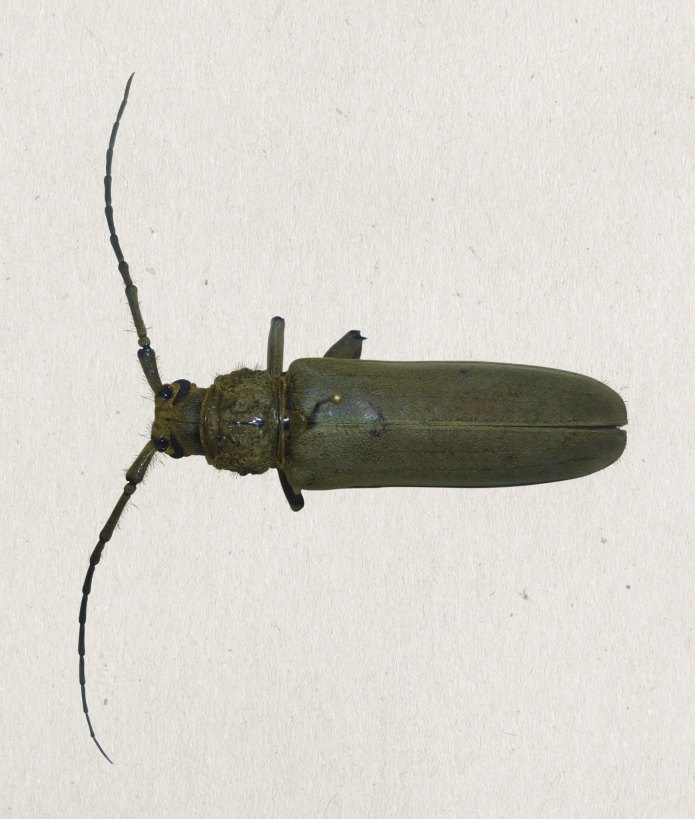
*Criodion cinereum* (Olivier, 1795) habitus of adult: dorsal view

Adults of *C. cinereum* differ from the similar South American *C. torticolle* Bates, 1870 by the basal antenommere not reaching the anterior margin of the pronotum, pronotal gibbosity scarecely apparent; prosternal process not exceeding the procoxae; and middle femoral apical spines not markedly projected (Olivier 1795, Gahan 1892). According to Martins (2004), the distinctions between *C. cinereum* and *C. torticolle* have always been problematic. (Fig. 1)

### Criodion tuberculatum

Gahan, 1892

#### Materials

- **Type status:** Other material. **Occurrence:** recordedBy: Roberto Cambra, Alonso Santos Murgas; individualCount: 1; sex: male; lifeStage: adult; **Taxon:** scientificName: *Criodion tuberculatum* Gahan 1892.; acceptedNameUsage: Criodion tuberculatum; parentNameUsage: Cerambycidae; originalNameUsage: Criodion tuberculatum; nameAccordingTo: Gahan, C.J. 1892. Notes on Longicorn Coleoptera of the group Cerambycinae, with descrption of new species. Ann. Mag. Nat. Hist. London, 6(9):25; namePublishedIn: Gahan, C.J. 1892. Notes on Longicorn Coleoptera of the group Cerambycinae, with descrption of new species. Ann. Mag. Nat. Hist. London, 6(9):19-32; higherClassification: "Animalia";"Arthropoda"; "Hexapoda"; "Insecta"; "Coleoptera"; "Cerambycidae";"Cerambycinae";"Spallotrichina"; "Criodion"; "Criodion tuberculatum"; kingdom: Animalia; phylum: Arthropoda; class: Insecta; order: Coleoptera; family: Cerambycidae; genus: Criodion; specificEpithet: tuberculatum; taxonRank: species; scientificNameAuthorship: Gahan, 1892; vernacularName: Longhorn beetle; nomenclaturalCode: ICZN; taxonomicStatus: Accepted; **Location:** higherGeographyID: TGN: 7005572; higherGeography: Central America, Panama, Darien, Parque Nacional Darien, Estacion Rancho Frio; continent: Central America; country: Panama; countryCode: PA; stateProvince: Darien; county: Parque Nacional Darién; municipality: Estación Rancho Frio; verbatimElevation: 545 m; minimumElevationInMeters: 0; maximumElevationInMeters: 545; verbatimCoordinates: 7°58'58.06"N, 77°44'19.72"W; **Identification:** identificationID: Criodion tuberculatum; identifiedBy: Alfredo Lanuza-Garay; dateIdentified: 2015-12-10; identificationReferences: "Notes on longicorn Coleoptera of the group Cerambycinae, with descriptions of new genera and species. Gahan 1892", "Cerambycidae Sul Americanos. Martins 2004"; identificationRemarks: Distinguished between *C. tuberculatum* and *C. rhinoceros* based on the comparative of the mandibles and the color of the body pilosity; **Event:** samplingProtocol: Malaise traps; eventDate: 2000-11-16; year: 2000; month: 11; day: 16; habitat: Semi-desciduous tropical forest; **Record Level:** type: Physicalobject; language: es; rightsHolder: Universidad de Panama; bibliographicCitation: Gahan, C.J. (1892).Notes on longicorn Coleoptera of the group Cerambycinae, with descriptions of new genera and species. The Annals and Magazine of Natural History 6 (9): 19‑32; institutionID: Museo de Invertebrados, Universidad de Panama; institutionCode: MIUP; collectionCode: Insects; ownerInstitutionCode: UP; basisOfRecord: Preservedspecimen

#### Distribution

Previously, *C. tuberculatum* had been recorded only in French Guiana, Brazil, and Peru (Gahan 1892, Monné 2015)

#### Notes

**Figure 2.**
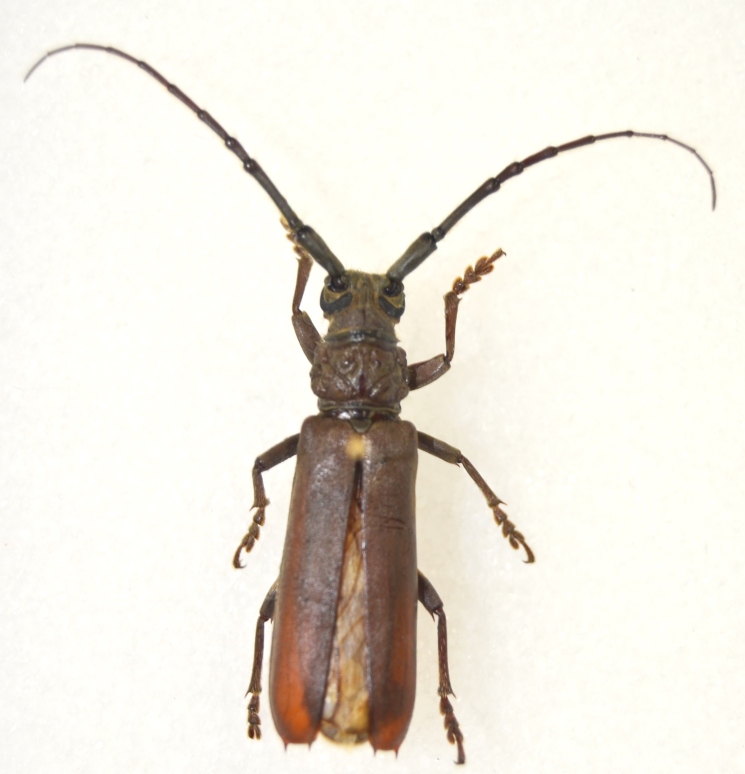
*Criodion tuberculatum* Gahan, 1892 habitus of adult: dorsal view

This species resembles *C. rhinoceros* Bates 1870 by the presence of two apical spines on each elytron, a character that separates these species from other species of *Criodion*. Characteristics such as *C. rhinoceros* having strong but unarmed mandibles, and the color of the body pilosity are very helpful in distinguishing the two species, being yellowish in *C. tuberculatum* and grayish in *C. rhinoceros*. (Fig. 2)

## Discussion

The biology and ecology of *Criodion* species are little known. According to Bondar (1921), Lorenzi (1992) and Martins (2004) *Criodion* larvae feed on plants belonging to the family Fabaceae. They eat live tissues of these plants and make two kinds of galleries; 1) twisted, with openings at several places along the trunk or at the base of the trunk and 2) straight, with exits at the basal end of the galleries. Some of their host plant genera are present in Panama, e.g., *Inga* (Guaba), *Acacia* (Cachito), and *Bauhinia* (Pezuña de Vaca) (Correa et al. 2004, Condit et al. 2013), but the host plants of *C. cinereum* and *C. tuberculatum* in Panama are unknown. Fiorentino and Bellomo (1997) report that some species of *Criodion* are parasitized by chalcid wasps (*Eumegalocus* sp., *Parastypiura* sp.), encyrtid wasps (*Dioencyrtus fiorentinoi*, *Amauroencyrtus micans*), and tachinid flies, however, the parasitoids of *C. cinereum* and *C. tuberculatum* in Panama are unknown.

## Supplementary Material

XML Treatment for Criodion cinereum

XML Treatment for Criodion tuberculatum
